# Robot-assisted laparoscopic antegrade versus open inguinal lymphadenectomy: a retrospective controlled study

**DOI:** 10.1186/s12894-019-0571-4

**Published:** 2019-12-23

**Authors:** Hualiang Yu, Yongliang Lu, Yi Xiao, Jiaxiang Guo, Xiaotao Yin, Yu Yang, Hongwei Wang, Jiangping Gao

**Affiliations:** 1 Department of Urology, The Fourth Medical Center of Chinese PLA General Hospital, 51th Fucheng Street, Haidian District, Beijing, 100048 China; 2 Department of Pathology, The Fourth Medical Center of Chinese PLA General Hospital, 51th Fucheng Street, Haidian District, Beijing, 100048 China

**Keywords:** Robot-assisted surgery, Penile cancer, Antegrade, Inguinal lymphadenectomy

## Abstract

**Background:**

To investigate the surgical methods and clinical results of robot-assisted laparoscopic antegrade inguinal lymphadenectomy.

**Methods:**

A retrospective study was performed on clinical data from 19 patients with penile cancer admitted from March 2013 to October 2017. Among them, nine patients underwent robot-assisted laparoscopic antegrade inguinal lymphadenectomy (robot-assisted group) and 10 patients underwent open inguinal lymphadenectomy (open group). In the robot-assisted group, preoperative preparation, patient position, robot placement, design of operating channel and establishment of operating space are described. Key surgical procedures and techniques are also summarized. In addition, the number of lymph nodes removed, postoperative complications and follow-up in both groups were statistically analyzed.

**Results:**

For the 9 patients in the robot-assisted group, surgery was successfully accomplished at 17 sides without intraoperative conversion to open surgery. The surgery time for each side was 45~90 min using laparoscope with an average of 68.5 ± 13.69 min/side. The intraoperative blood loss was estimated to be < 10 ml/side, and the number of removed lymph nodes was not significantly different from that of the open group (12 ± 4.2/side vs.11 ± 5.8/side, *P* = 0.84). There were no postoperative complications such as skin necrosis, delayed wound healing and cellulitis in the robot-assisted group. Skin-related complications occurred in 9 (45%) of the 20 sides in the open group. During a median follow-up of 25 months in robot-assisted group and 52.5 mouths in open group, was not significantly different there were no statistical differences in recurrence-free survival between the groups (75% vs 60%, *p* = 0.536).

**Conclusion:**

Robot-assisted laparoscopic antegrade inguinal lymphadenectomy achieved the desired surgical outcomes with fewer intraoperative and postoperative complications. The robotic arms of the surgical system were placed between the lower limbs of each patient. There was no need to re-position the robotic arms during bilateral inguinal lymphadenectomy. This simplified the procedure and reduced the use of trocars. If necessary, pelvic lymphadenectomy could be performed simultaneously using the original trocar position.

## Background

Penile cancer is a relatively rare genitourinary malignancy. Regional lymph node metastasis is considered to associate closely with the patient prognosis. Patient 5-year survival rate without regional lymph node metastasis is 95 to 100%, and drops to 50% for patients with multiple inguinal lymph node metastasis [1]. Hence, inguinal lymphadenectomy is an important treatment strategy for penile cancer. The efficacy of open inguinal lymphadenectomy is definite, however the incidence rates of postoperative complications such as skin necrosis and delayed wound healing are about 50% [2, 3]. The use of laparoscopic technology is compromised due to its own operational limitations [4, 5]. The da Vinci robotic system can provide stable three-dimensional images for surgeons, with high precision and excellent flexibility. Many of the shortcomings of simple laparoscopic surgery are addressed using the da Vinci robotic system. Da Vinci robot-assisted laparoscopic antegrade inguinal lymphadenectomy was successfully performed in nine patients from August 2016 to October 2017, obtaining satisfactory efficacy, which we report in this study.

## Methods

### General characteristics

The robot-assisted group had nine patients with penile cancer with an average age of (50.0 ± 7.17) years old, ranging from 40 to 62 years old. Their body mass index was (27.3 ± 3.93) kg/m^2^, ranging from 21.67 kg/m^2^ to 33.21 kg/m^2^. The open surgery group had ten patients with an average age of (54.9 ± 13.12) years old, ranging from 24 to 68 years old. Their body mass index was (27.0 ± 2.53) kg/m^2^, ranging from 22.03 to 30.03 kg/m^2^. All nine patients in the robot-assisted group underwent treatment for penile cancer with pathological diagnosis of primary lesions being all squamous cell carcinoma. This included three patients with well-differentiated squamous cell carcinoma, four with moderately differentiated squamous cell carcinoma, and two with poorly differentiated squamous cell carcinoma. Of the nine patients, six had subcutaneous connective tissue invasion (T1 stage) and three had corpus spongiosum urethrae infiltrate (T3 stage). Preoperative and assistant trocar examinations suggested that there were inguinal lymphadenectasis, but no pelvic lymphadenectasis. One patient was cN1 stage, five were cN2 stage, and three were cN3 stage. None of the patients had distant metastasis (M0 stage). All patients had surgical indicators for inguinal lymphadenectomy. Based on preoperative evaluation and intraoperative pathology of frozen sections, six patients simultaneously underwent robot-assisted laparoscopic pelvic lymphadenectomy using the original trocar position. In the open group, eight of the ten patients were T1 stage, one was T2 stage and one was T3 stage. The preoperative clinical N stage were as follows, cN1 in two patients, cN2 in seven and cN3 in one (Table 1).

### Surgical procedure

The open inguinal lymphadenectomy was performed via the (inverse) S-shaped inguinal incision. While the robot-assisted surgical procedure was as follows.

### Patient and trocar position

**Fig. 1:**
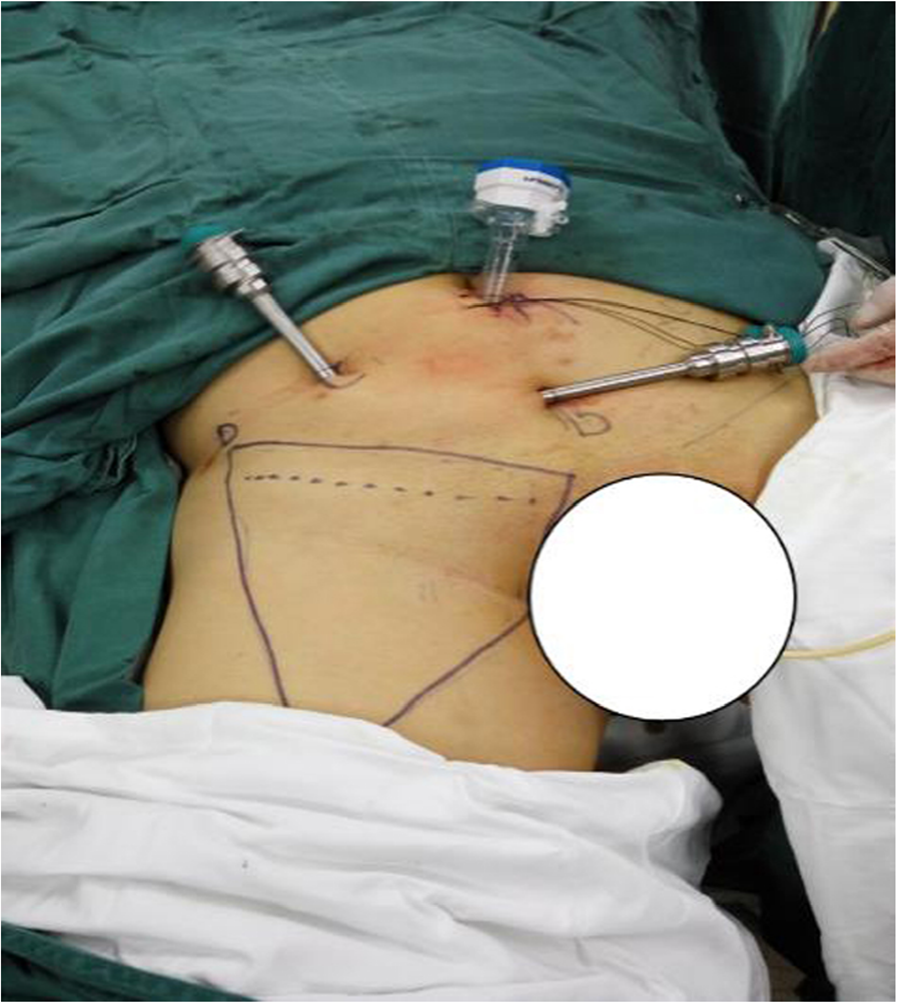
Position of the Trocar during robot-assisted laparoscopic antegrade inguinal lymphadenectomy

General anesthesia was administered to patients. Patients were in the supine position with the head 15° lower compared to the hip. Both lower extremities were straightened and abducted by about 45° (scissor position). Both of the knee joints were slightly flexed and rotated externally, with the catheter indwelled. The bedside robotic arm of the da Vinci robot was pushed between the two legs of the patient. A 2 cm longitudinal incision was made at the lower margin of the umbilicus. The skin, adipose layer of superficial fascia (Camper fascia) and membranous layer of superficial fascia (Scarpa fascia) were incised. Blunt dissection was performed using the index finger on the surface of the external oblique aponeurosis, and subcutaneous space was established by expansion of self-made balloon. A 12 mm trocar was placed via the incision to be used as a lens hole. Subcutaneous pneumoperitoneum space was created using CO_2_, with pressure maintained at 12 mmHg (1 mmHg = 0.133 kPa). During right inguinal lymphadenectomy, a metal trocar of arm-2 was placed at the midpoint between the umbilical cord and pubis, subsequently a metal trocar of arm-1 was placed at the midpoint between the umbilical cord and right anterior superior iliac spine. A 12 mm trocar was then placed under arm-1 outside the metal trocar as an assistant trocar (Fig. 1). During left inguinal lymphadenectomy, the lens hole was unchanged, and the original trocar of arm-2 was used as the trocar of arm-1. Afterwards, a metal trocar of arm-2 was placed at the midpoint between the umbilical cord and the right anterior superior iliac spine.

**Table 1:** Demographic and Clinical Characteristics of Patients

	robotic group(*n* = 9)	open group(*n* = 10)
Age	50.0 ± 7.17 (40–62)	54.9 ± 13.12 (24–68)
BMI (kg/m^2^)	27.3 ± 3.93 (21.67–33.21)	27.0 ± 2.53 (22.03–30.03)
pT stage pT1/ pT2/ pT3	6/0/3	8/1/1
cN stage cN0/cN1/cN2/cN3	0/1/5/3	0/2/7/1

### Surgical procedure

**Fig. 2:**
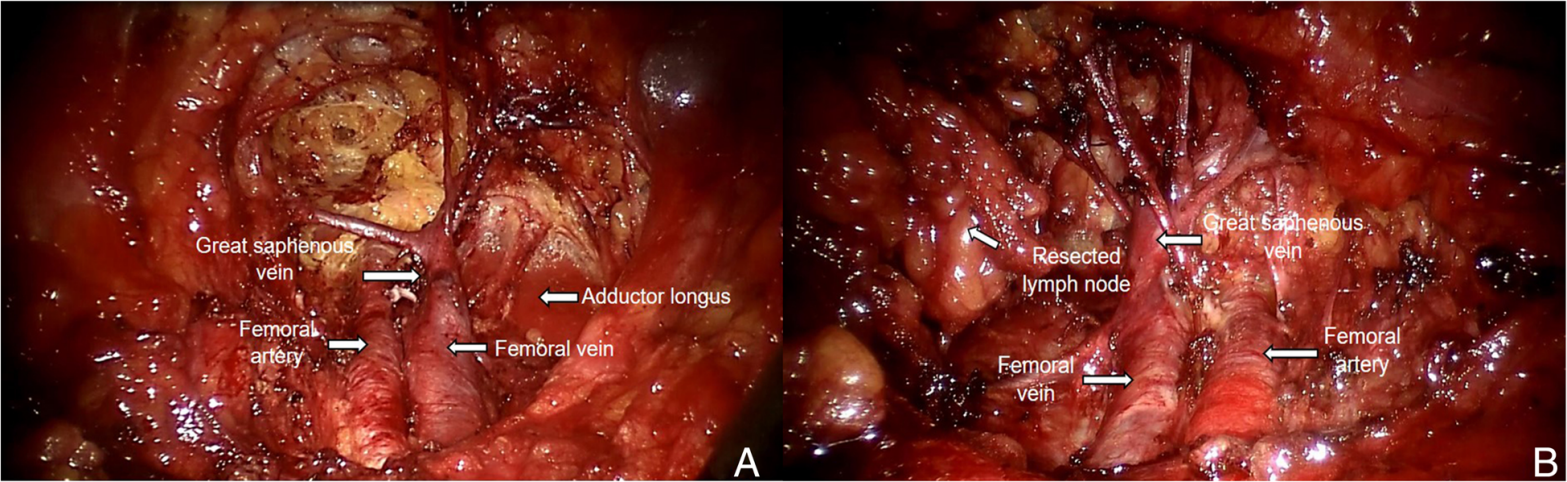
Robot-assisted laparoscopic antegrade inguinal lymphadenectomy. **a**. Resection of inguinal lymph nodes on the left side. **b**. Resection of inguinal lymph nodes on the right side

1. *Inguinal lymphadenectomy:* The range of inguinal lymphadenectomy was 2 cm above the inguinal ligament as the upper boundary, apex of the femoral triangle as the lower boundary, medialis sartorius muscle as the outer boundary, and lateralis of the long adductor muscle as the inner boundary. First, dissociation was performed from the surface of the external oblique aponeurosis to the superficial layer in order to remove adipose tissues and superficial lymph nodes in the superficial fascia. Then pressure was applied to the inguinal ligaments by hand and surface projection of the femoral blood vessel was performed. The spermatic cord was pulled to help localization during surgery. The fascia lata under the inguinal ligament was exposed and the cribriform fascia was cut open while the femoral artery and vein were exposed to let the surface skeletonize. The femoral vein was dissociated downward to expose the great saphenous vein and its branches, which were retained. The dissociation was continued until the apex of the femoral triangle. Then, the lateralis of the long adductor muscle was exposed on the medial side of the femoral vein. The medialis sartorius muscle was then exposed on the lateral side of the femoral artery. The femoral nerves were protected, and adipose tissue as well as deep lymph nodes in the femoral canal were removed from the assistant trocar and placed in a specimen bag for pathological examination (Fig. 2). After completing the surgery, hemostasis was performed thoroughly on the wound and a negative-pressure drainage tube was placed via the assistant trocar. Puncture holes were then sutured. The opposite side was treated in the same way.

Pathology examination of frozen sections was performed for the nine patients during surgery. Results from five patients showed that the number of positive lymph nodes in the unilateral groin was ≥2. Preoperative examination of one patient showed that there was recurrence of lymph node metastasis in the right groin and fused nodular enlargement of about 4.5 cm × 4.3 cm × 3.0 cm. Hence, pelvic lymphadenectomy was performed via the original trocar position for the above six patients.

2) *Pelvic lymphadenectomy:* Lymph nodes in the bilateral iliac crests, external iliac, internal iliac and obturator were removed. After bilateral inguinal lymphadenectomy, the docking of the robotic arm and the corresponding trocar were released and the arms were temporarily closed. However, the entire system was not moved. At the position of the original lens hole, a veress needle or a 12 mm trocar was punctured into the abdominal cavity to establish pneumoperitoneum with pressure maintained at 14 mmHg. The trocar position of arm-1 in the right inguinal lymphadenectomy and trocar position of arm-2 in the left inguinal lymphadenectomy was used as the positions of arm-1 and arm-2 for pelvic lymphadenectomy. 8 mm trocars were placed at the corresponding puncture points. 12 mm trocars were placed under arm-1 or arm-2 as the assistant trocar to dock robotic arms. Subsequently, monopolar electrosurgical and bipolar electrosurgical scissors were used to remove pelvic lymph nodes, respectively.

### Postoperative treatment

After surgery, the groin area was bandaged with elastic. Patients were asked to lie in bed with limited movement and have a low-fat high-protein diet. The drainage tube was removed if drainage volume at 24 h was less than 40 ml. Patients were regularly followed-up at the outpatient department after discharge or continued follow-up treatment.

## Results

**Fig. 3:**
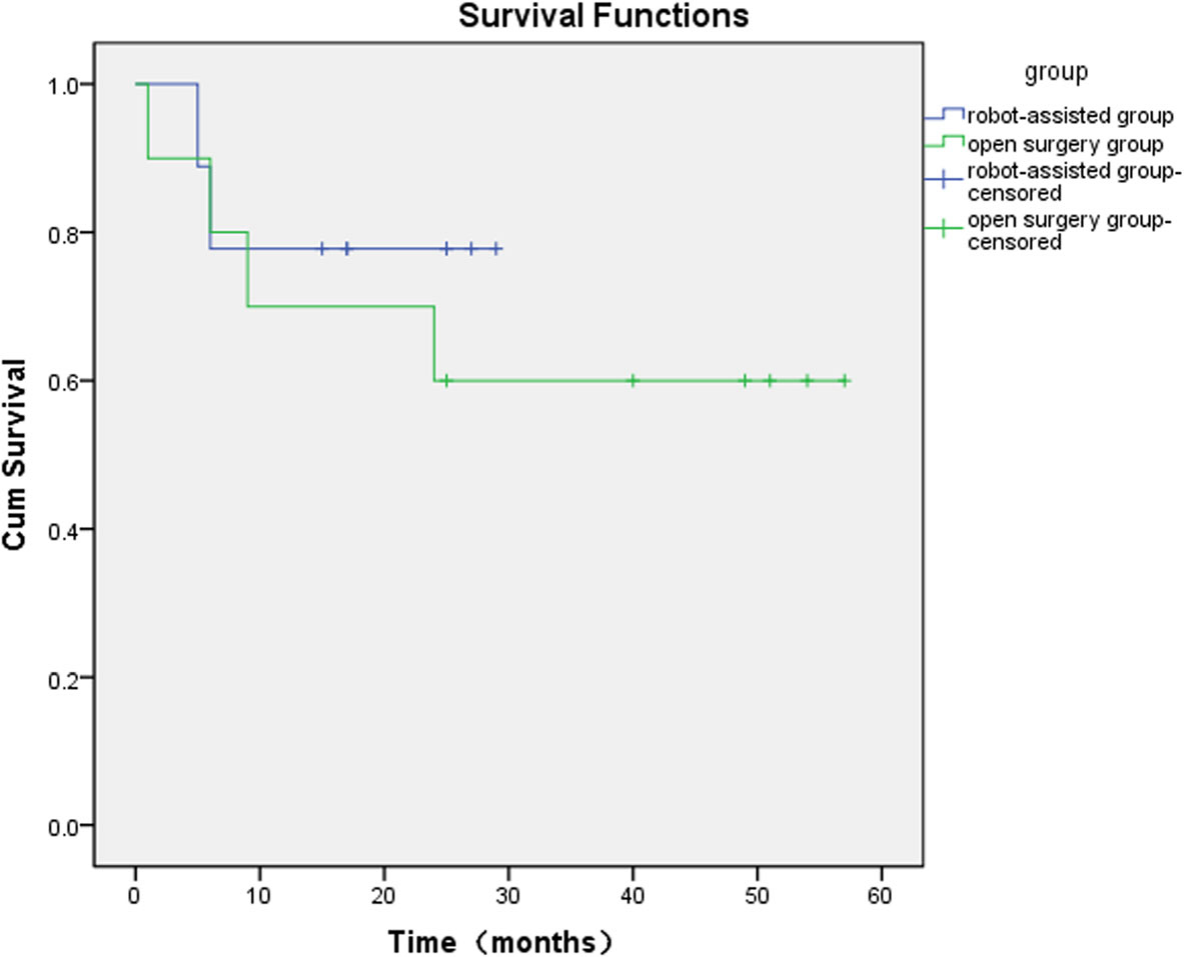
Kaplan-Meier survival curve of two groups

For the nine patients in the robot-assisted group, surgeries for all the 17 sides were successful without intraoperative conversion to open surgery. The operation time was (68.5 ± 13.69) min/side under laparoscope; the intraoperative blood loss was < 10 ml/side, and (12 ± 4.0) lymph nodes in the left side were removed, ranging from 7 to 18. A total of (12 ± 4.0) lymph nodes in the right side were removed, ranging from 5 to 21, with the average of (12 ± 4.2) node/side. For the ten patients in the open-surgery group, surgeries were successful for all the 20 sides. The average number of lymph nodes removed was (11 ± 5.8) per side, ranging from 2 to 27, which was not statistically different compared to the robot-assisted group (*P* = 0.84). The postoperative pathological N staging of the robotic-assisted group were pN0, pN1, pN2, pN3 in 2, 2, 4 and 1 patients, and the open group were pN0, pN1, pN2 in 1, 1, and 8 patients. For the 20 sides in the open surgery group, 9 sides (45%) had skin-related complications., including skin necrosis, wound inflammatory exudation and cellulitis with different severity. When necessary, the negative pressure suction or flap transplantation were conducted for the infected or non-healing wound. In the robot-assisted group, stitches were removed for all nine patients around 7 days post-surgery. There were no complications such as skin necrosis, delayed wound healing and cellulitis. The lymphatic complications of two groups occurred slightly, which had a good outcome after proper treatment. In the open group, lymphorrhagia occurred in 4 cases, and lower limbs edema occurred occasionally in 1case after too much physical work. In the robot-assisted group five patients had lymphatic leakage in the inguinal region, which resolved after appropriate treatments such as pressurization and drainage. Lymphocoele occurred in one post-discharge patient, which was cured by puncture and continuous drainage. No genitalia lymphedema was observed in both groups. In the robot-assisted group, nine patients were followed up for 15–29 months with a median follow-up time of 25 months. However, one patient was lost for follow-up. Two patients developed recurrence and metastasis of the pelvic and abdominal cavities at five to 6 months post-surgery and died of tumor progression. The remaining six patients had no recurrence and metastasis. The recurrence-free survival rate was 75%. In the open surgery group, ten patients were followed up for 25–70 months with a median time of 52.5 months. Of them, four patients died of tumor recurrence and metastasis and the remaining 6 patients had no recurrence and metastasis. The recurrence-free survival rate was 60%, and there were no statistical differences between the groups (*P* = 0.536) (Fig. 3) (Table 2). Of the two groups, all the six cases with recurrence or metastasis had a staging of pN2 or pN3, while other 14 cases with pN0 or pN1 had no recurrence and metastasis. All the six patients, dying of tumor progression, developed inguinal and pelvic lymph nodes recurrence or metastasis, leading to extensive abdominal metastasis with cancer cachexia. In summary, from the number of lymph nodes removed by surgery and the follow-up status, robotic-assisted laparoscopic antegrade inguinal lymphadenectomy was a safe and effective surgical method and was superior to traditional open surgery in terms of postoperative complications.

**Table 2:** Intra- and Post-Operative Characteristics of Robotic and Open Groups

	robotic group (N = 9,17 sides)	open group (N = 10, 20 sides)	p
Operation time/side (min)	68.5 ± 13.69	Null	
Blood loss /side (ml)	< 10	Null	
Lymph nodes/side	12 ± 4.2(5–21)	11 ± 5.8 (2–27)	0.84
pN stage pN0/pN1/pN2/pN3	2/2/4/1	1/1/8/0	
Complication	0/17	9/20	
Skin-related (side)	5/9	4/10	
Lymphorrhagia (case number)	1/9	0/10	
Lymphocoele (case number)	I, 8	I, 5	
Clavien classification	IIIa,1	IIIa, 4 IIIb, 1	
Follow up			0.536
No recurrence	6	6	
Recurrence	2	4	
Loss to follow-up	1	0	

## Discussion

Penile cancer is a relatively rare malignancy. Due to differences in religious beliefs and health habits, the incidence of penile cancer varies significantly, with incidences of 0.1–0.9/100,000 in Europe and the United States and 19/100,000 in some economically underdeveloped regions in Asia, Africa and South America [6, 7]. Penile cancer spreads via lymph node metastasis, and first occurs in the inguinal lymph nodes. Lymph node metastasis is considered to be closely associated with the prognosis of penile cancer. Hence, the prognosis of penile cancer is not only associated to the stage and grade of the primary tumor, but also the presence of metastasis, degree of metastasis, lymph node dissection and timing of lymph node dissection [8]. The 5-year survival rate of patients after preventive lymph node dissection can reach 80 to 90%, while the 5-year survival rate is only 30 to 40% for patients who undergo surgery after lymph node metastasis [9]. For patients with inguinal lymphadenectasis, antibiotic treatment is usually recommended to eliminate inflammatory lesions. However, since the European Association of Urology Guidelines 2014 edition, the recommendation is as follows: With uni- or bilateral palpable inguinal lymph nodes (cN1/cN2), metastatic lymph node disease is highly likely. The notion that these may be inflammatory and that antibiotic treatment should first be used is unfounded and dangerous as it delays curative treatment. Palpably enlarged groin lymph nodes should be surgically removed, pathologically assessed (by frozen section) and, if positive, a radical inguinal lymphadenectomy should be performed. In clinically doubtful cases, US-guided fine needle aspiration cytology is an option. For patients with cN0 primary tumor stage >T1G2, bilateral modified inguinal lymphadenectomy (mILND) or dynamic sentinel node biopsy (DSNB) is recommended [10].

The range of the inguinal lymphadenectomy was constituted as 2 cm above the inguinal ligament as the upper boundary, apex of the femoral triangle as the lower boundary, medialis sartorius muscle as the outer boundary, and lateralis of long adductor muscle as the inner boundary [8]. Open surgery can completely resect regional lymph nodes, however the subcutaneous exfoliation surface is wide, the blood supply to the skin margin is poor, and the incidence of postoperative skin flap necrosis as well as delayed healing are as high as 50% [11, 12]. Extended hospitalization stay and wound care are required after open surgery. In 2006, Tobias-Machado et al. reported on laparoscopic inguinal lymphadenectomy for the first time. Its feasibility was primarily proved by comparison with open surgery. Laparoscopic inguinal lymphadenectomy had the same efficacy for tumor control compared to traditional open surgery. However, the overall incidence rate of postoperative complications decreased significantly, and there were no complications related to skin incision, such as skin flap necrosis and non-healing wound. Postoperative recovery was rapid compared to open surgery [11–13]. In 2015, Liu CE et al. obtained similar results in a systematic review of the safety and feasibility of video endoscopic inguinal lymphadenectomy for vulvar cancer [12]. With the gradual development of video endoscopic inguinal lymphadenectomy, several surgeons tried to perform the surgery under a single-port laparoscope. However, due to limitations in surgical instruments and operation methods, the surgery time was long and required extensive surgical expertise. Moreover, vascular complications were difficult to be treated and the surgical technique had a high learning curve [14].

In the first reported case of robot-assisted laparoscopic inguinal lymphadenectomy (RAVEIL) by Josephson et al. in 2009, as well as surgical cases reported by Sotelo R et al. in 2013 and Ma Jiajia in 2014, the great saphenous vein and its branches were precisely separated and retained, which reduced vascular injury and had a more thorough dissection. The flexibility and precision of robots can better handle the possible complications during surgery [15–17]. In previous RAVEIL, the robotic arms are usually pushed from the side of the body, and retrograde dissection was conducted from the apex of the femoral triangle [18]. In addition, during surgery, the robotic arms had to be moved to clear inguinal lymph nodes in the opposite side or in pelvic lymph nodes. In this study, the robotic arms were placed between the legs of the patients and antegrade dissection was performed from the top of the inguinal ligament downward. The advantages of this method are summarized as follows: ① There was no need to move the robotic arm during bilateral inguinal lymphadenectomy because the robotic arm were placed between the lower limbs, which simplified the surgical steps, reduced the number of trocars and decreased surgery time. ② During surgery, based on pathological results of frozen sections, pelvic lymphadenectomy could be performed simultaneously using the original skin incision with the patient’s position and position of the robotic arm not shifting. ③ During surgery, inguinal ligament, spermatic cord, femoral artery and vein were used as markers for antegrade separation of the great saphenous vein and its branches. This reduced intraoperative vascular injury and postoperative complications. ④ Space between the robotic arms was larger, hence a greater degree of freedom and flexibility, which was conducive to intraoperative surgeries.

With regards to the nine patients in the robot-assisted group, robotic assisted endoscopic antegrade inguinal lymphadenectomy was performed for 17 sides. The experience gathered is summarized as follows: ① Layers were selected correctly to establish pneumoperitoneum. Various layers of the skin and the superficial fascia (adipose layer, membranous layer) were cut open. Then, sharp and blunt dissections were used until external oblique aponeurosis. ② The initial surgical space was fully established. Blunt dissection was performed exclusively using the index finger on the surface of the external oblique muscle to ensure that arm-1 and arm-2, as well as the corresponding instruments could be placed. After docking the robotic arms, separation was continued under the endoscope to the position of the assistant trocar. ③ Surface projection of the femoral artery was marked before pneumoperitoneum was achieved. Before dissecting the lymph nodes, the testicle and the spermatic cords were pulled and the surface projection of the femoral artery was pressed. This was conducive to intraoperative localization for the surgeons. Inguinal ligament, spermatic vessels, femoral artery and femoral vein were exposed in sequence, which avoided vascular injury. ④ The superficial and deep lymph nodes were resected and boundary markers were exposed. Complying the principle of en bloc resection, ligation was preferred for disconnected lymphatic vessels, while electrocoagulation was the second option.

## Conclusions

In summary, compared with traditional open surgery, robot-assisted laparoscopic antegrade inguinal lymphadenectomy had the same efficacy on tumor control for the treatment of penile cancer. However, it was safer and had fewer postoperative wound complications. Currently only a few patients undergo this type of surgery and its efficacy and safety needs to be evaluated with more clinical data and longer-term follow-ups.

## Data Availability

All data generated or analysed during this study are included in this published article.

## Abbreviations

CO2: carbon dioxide
DSNB: dynamic sentinel node biopsy
mILND: modified inguinal lymphadenectomy
RAVEIL: robot-assisted laparoscopic inguinal lymphadenectomy
